# Supplementary material for: Atmospheric pressure mass spectrometric imaging of live hippocampal tissue slices with subcellular spatial resolution
Source: Nat Commun. 2017 Dec 13;8:2113. doi: 10.1038/s41467-017-02216-6 (PMC5727394; doi:10.1038/s41467-017-02216-6)
Supplement: Supplementary file 1 — Supplementary Information [file 41467_2017_2216_MOESM1_ESM.pdf]

Supplementary Figures

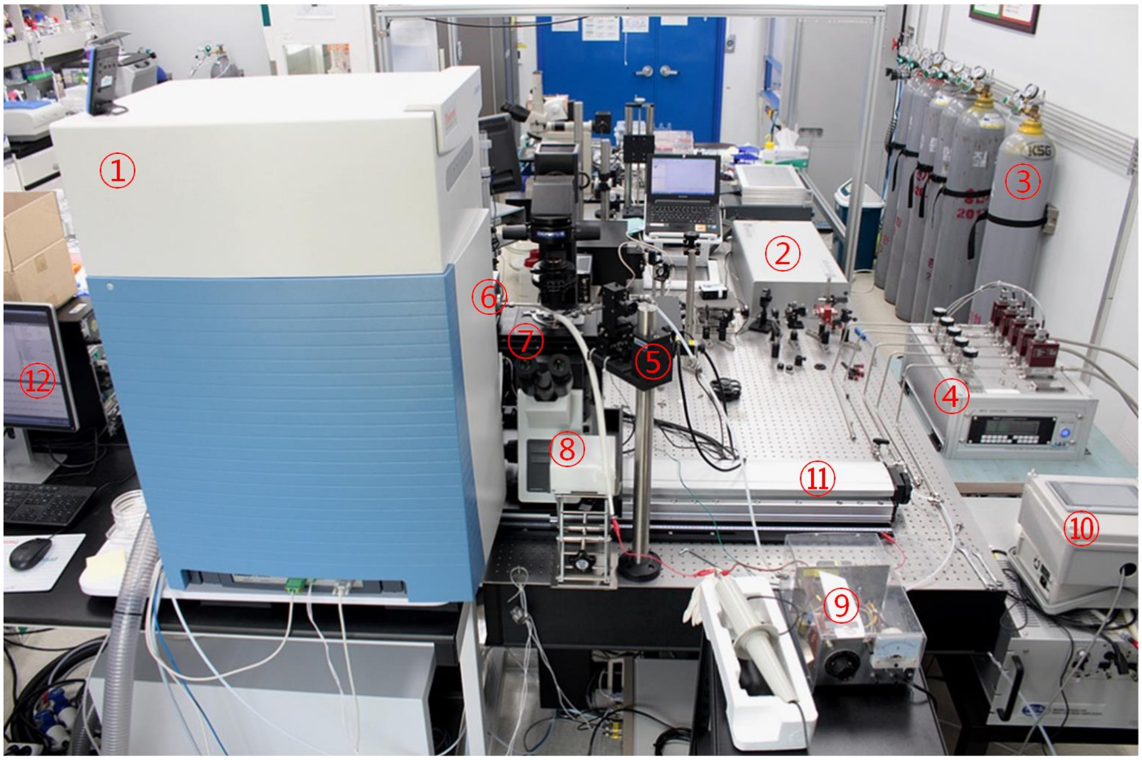

Supplementary Fig. 1 – A picture of the whole system for the AP-nanoPALDI mass spectrometry imaging employed in this study. The proposed AP-nanoPALDI MS system mainly consisted of mass analyzer, sampling stage, fs lasers system, atmospheric pressure plasma system, and airflow-assisted ion transport equipment; ① QE Orbitrap mass spectrometer, ② fs laser system, ③ gas cylinders, ④ 5 ch. gas flow controller, ⑤ nonthermal atmospheric pressure plasma jet, ⑥ airflow-assisted ion transfer system, ⑦ auto scanning stage (sample stage), ⑧ inverted microscope, ⑨ HV driving circuit and HV probe, ⑩ joystick of auto scanning stage, ⑪ long drive scope stage for microscope shift, and ⑫ PC for microscope imaging monitor, scanning software, and mass analysis software.

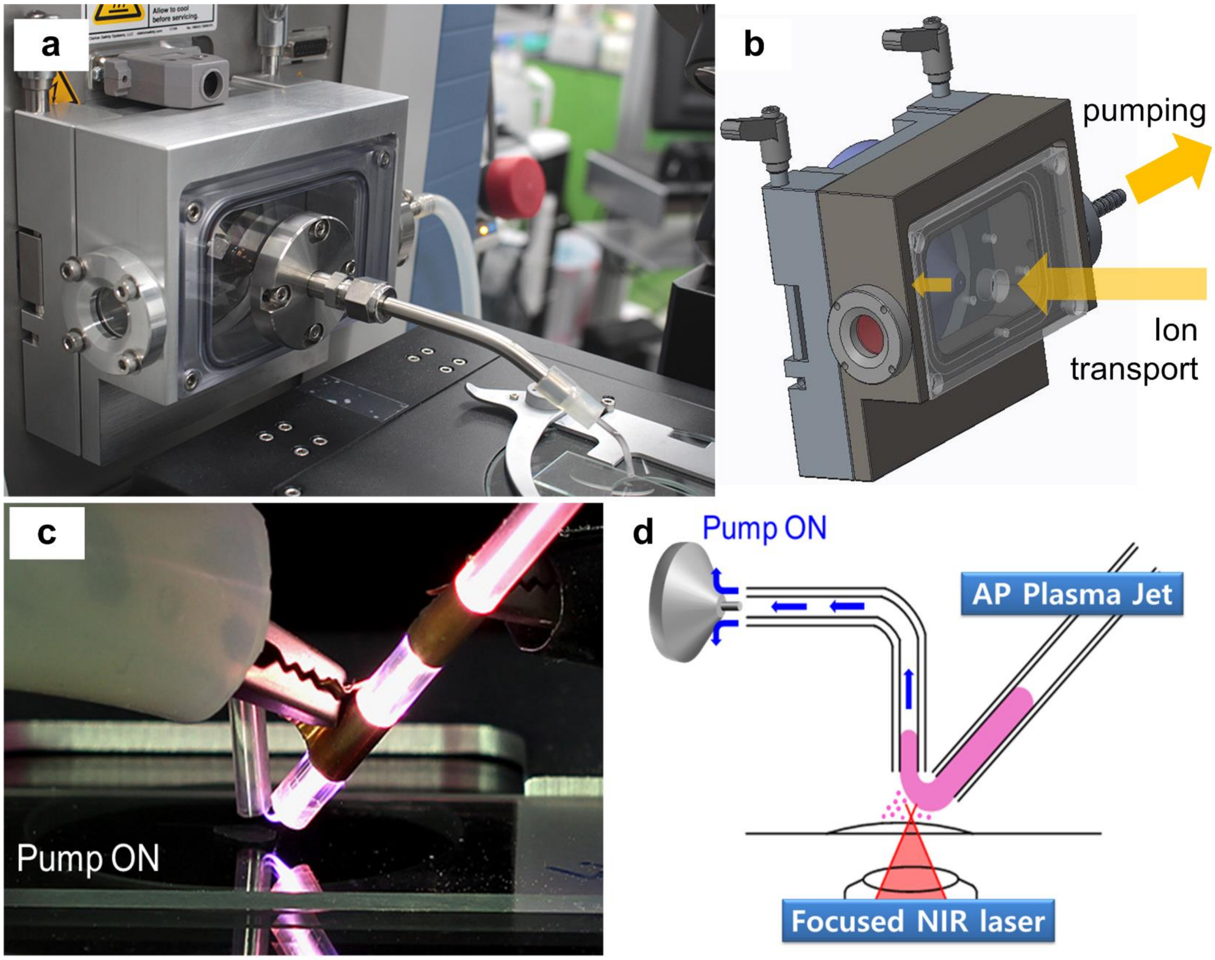

Supplementary Fig. 2 (**a**) Airflow-assisted ion transfer equipment consists of ion transfer tubing, chamber, and dry pump. (**b**) A drawing of the customized chamber. The pump was connected to the side hole of the chamber and generated the airflow toward the MS inlet inside the ion transfer tube. (**c**) A picture and (**d**) schematics during MS analysis on sampling stage. These show the configuration of atmospheric pressure desorption and ionization sources, sampling stage, ion transfer, and mass analyzer. Focused fs NIR lasers desorbed small substances from the sample, and then the nonthermal atmospheric pressure helium plasma jet ionized some of these substances. By the airflow-assisted ion transport equipment, the generated ions were induced to the MS inlet.

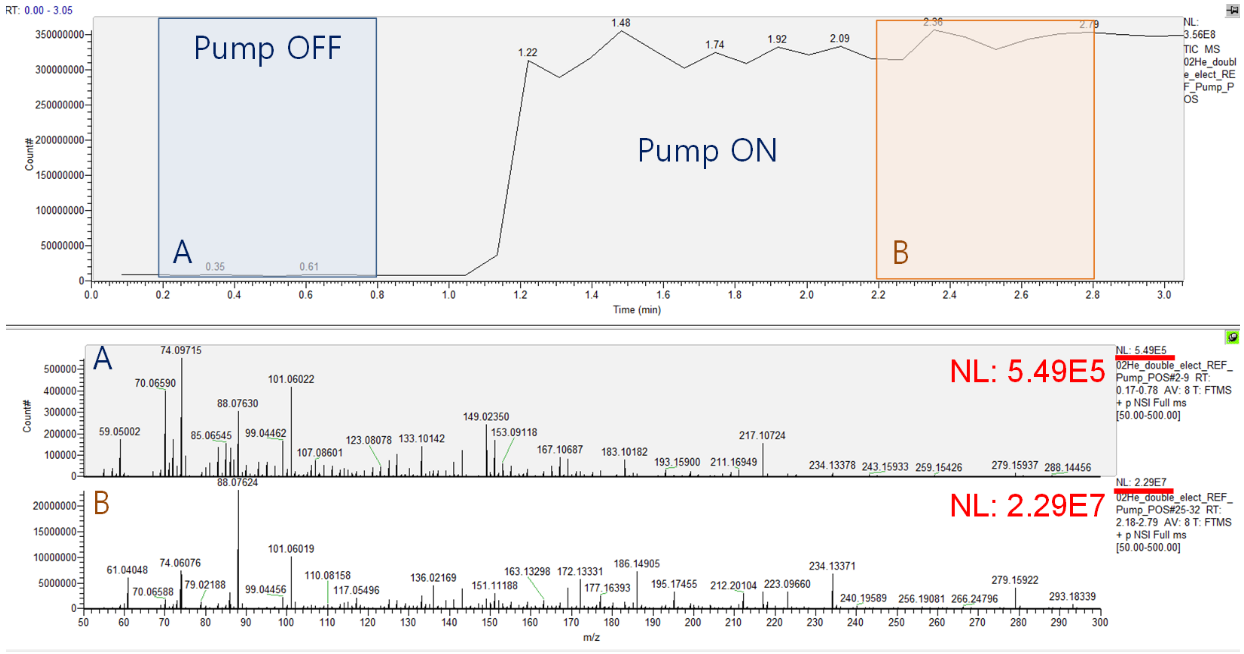

Supplementary Fig. 3 Performance of airflow-assisted ion transport equipment. Upper graph represented total ion currents (TIC) on MS software before and after the pump worked, which was installed by airflow-assisted ion transport equipment. The lower two graphs represented MS spectra before (A) and after (B) the pump worked. The intensity of total ions was observed to have a 40-fold increase compared to no pump condition.

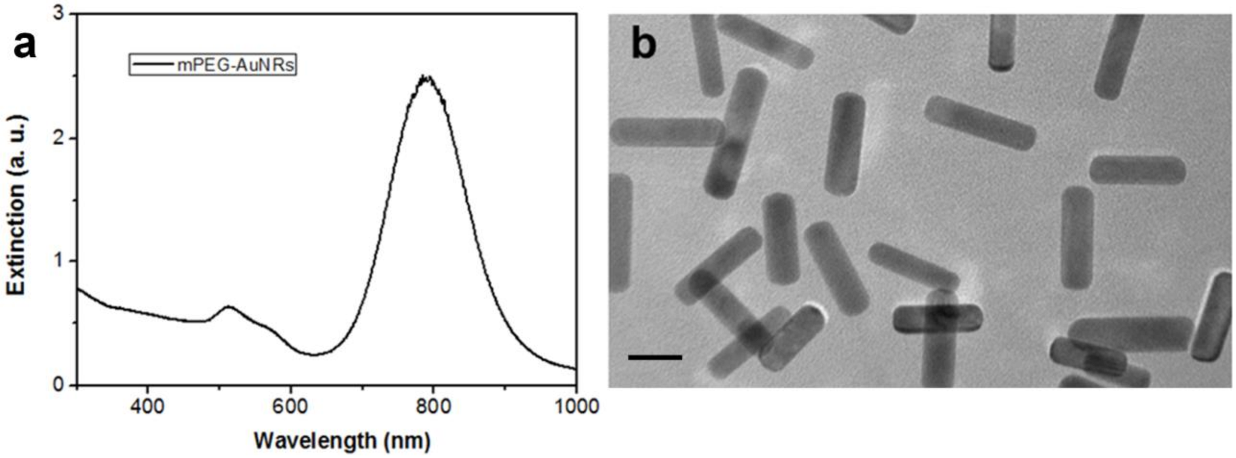

Supplementary Fig. 4 Optical characterization of mPEG-AuNRs; (**a**) UV-Vis spectra of mPEG-AuNRs in distilled water. (**b**) HR-TEM imaging of mPEG-AuNRs. Scale bar, 20 nm.

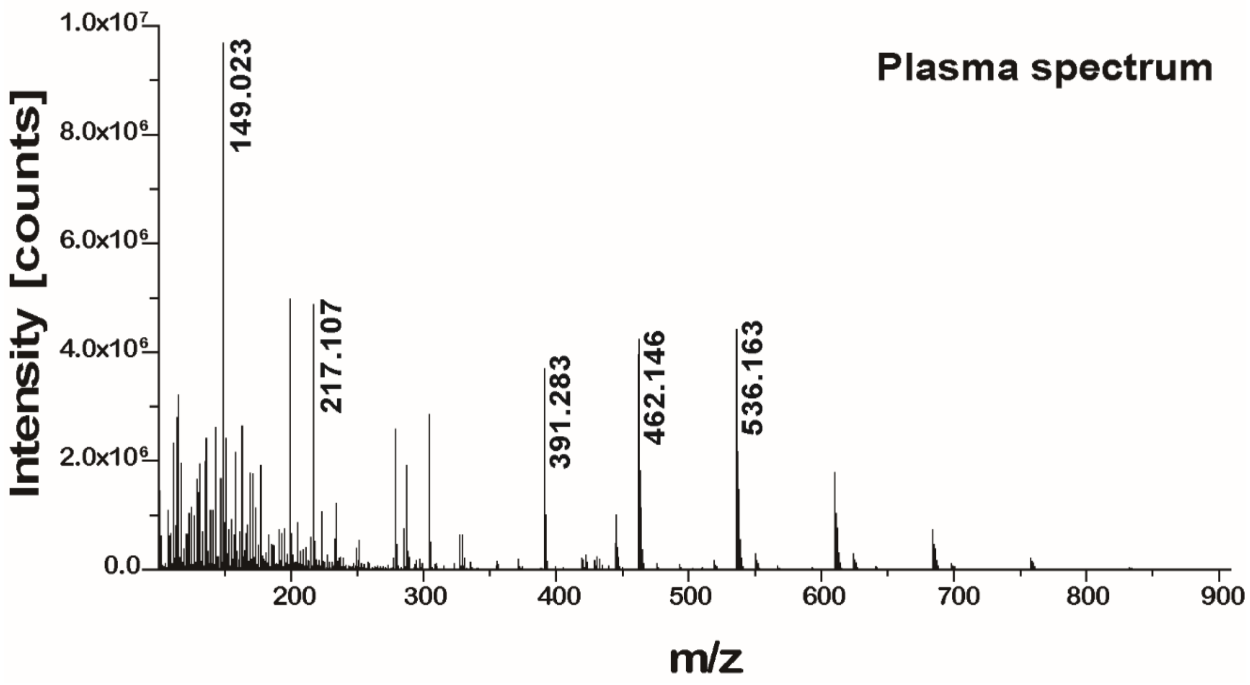

Supplementary Fig. 5 A positive ion mass spectrum recorded from nonthermal atmospheric pressure helium plasma using the AP-nanoPALDI method.

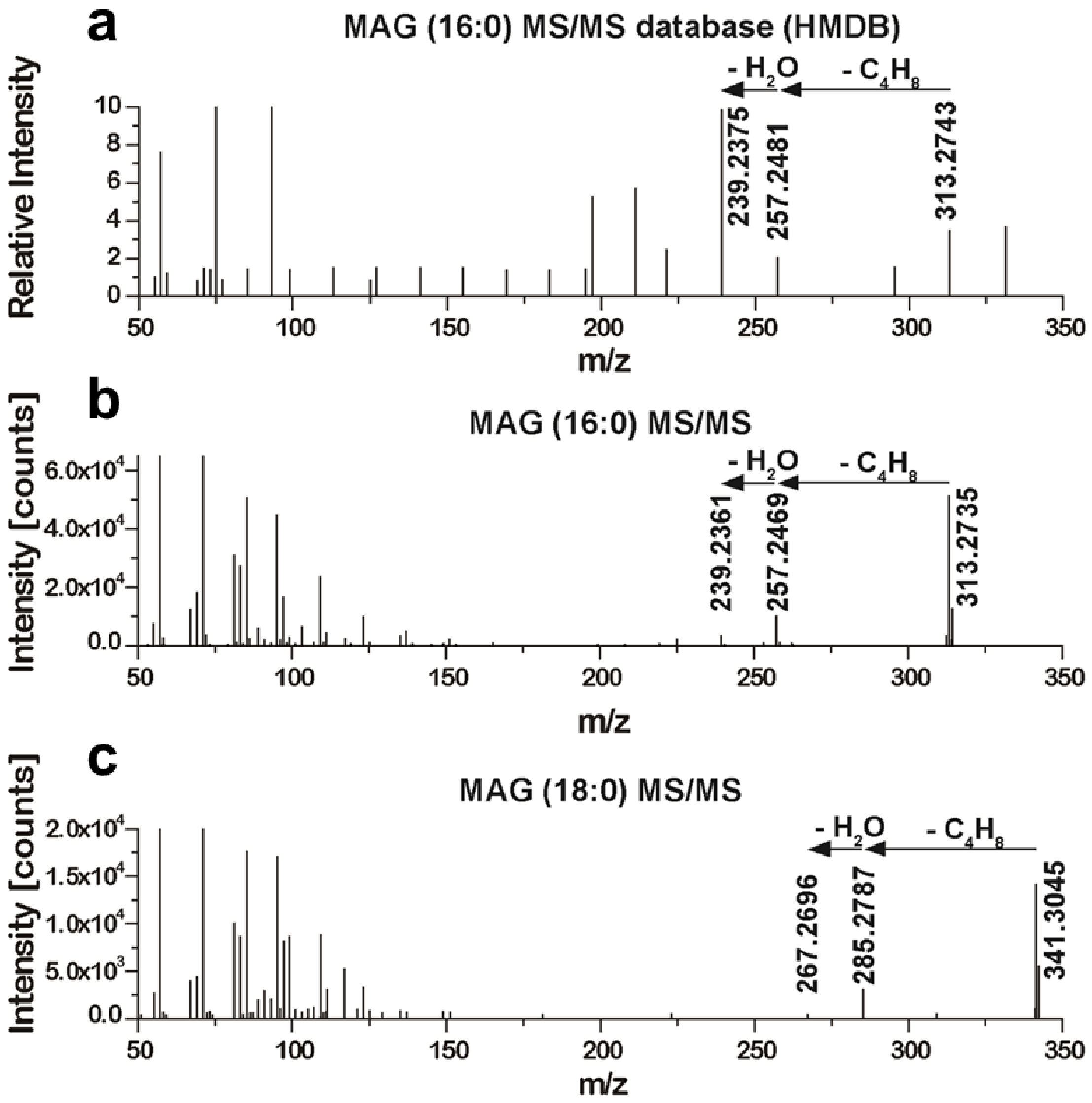

Supplementary Fig. 6 AP-nanoPALDI MS/MS data of saturated MAG ions from the mouse hippocampal tissue slice. (**a**) MS/MS data of MAG (16:0) from online MS/MS database searched with The Human Metabolome Database (HMDB, www.hmdb.ca) and AP-nanoPALDI MS/MS of (**b**) compound at m/z = 313.273 assigned to MAG (16:0) in the hippocampal tissue slice, and (**c**) compound at m/z = 341.305 assigned to MAG (18:0) in the hippocampal tissue slice. The saturated MAG ions were shown to have distinctive fragmentation patterns (-C_4H_8, -H_2O).

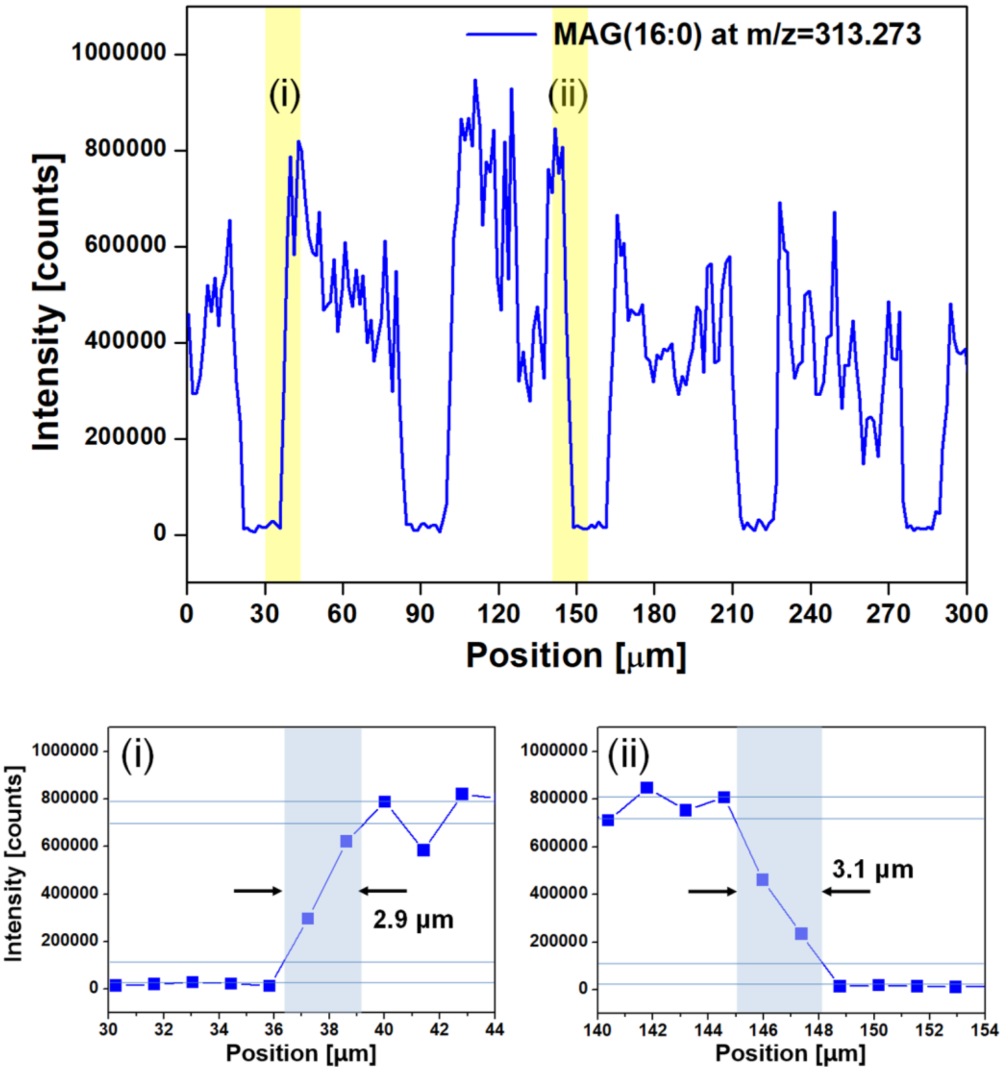

Supplementary Fig. 7 The x-line scan for MAG (16:0) ion at m/z = 313.273 measured by the hippocampal tissue slice on a 400 mesh grid with the sample moving velocities of 10 μm s^{-1} (**Fig. 5c** in the manuscript) and enlarged graphs of (i) the rising slope and (ii) the falling slope.

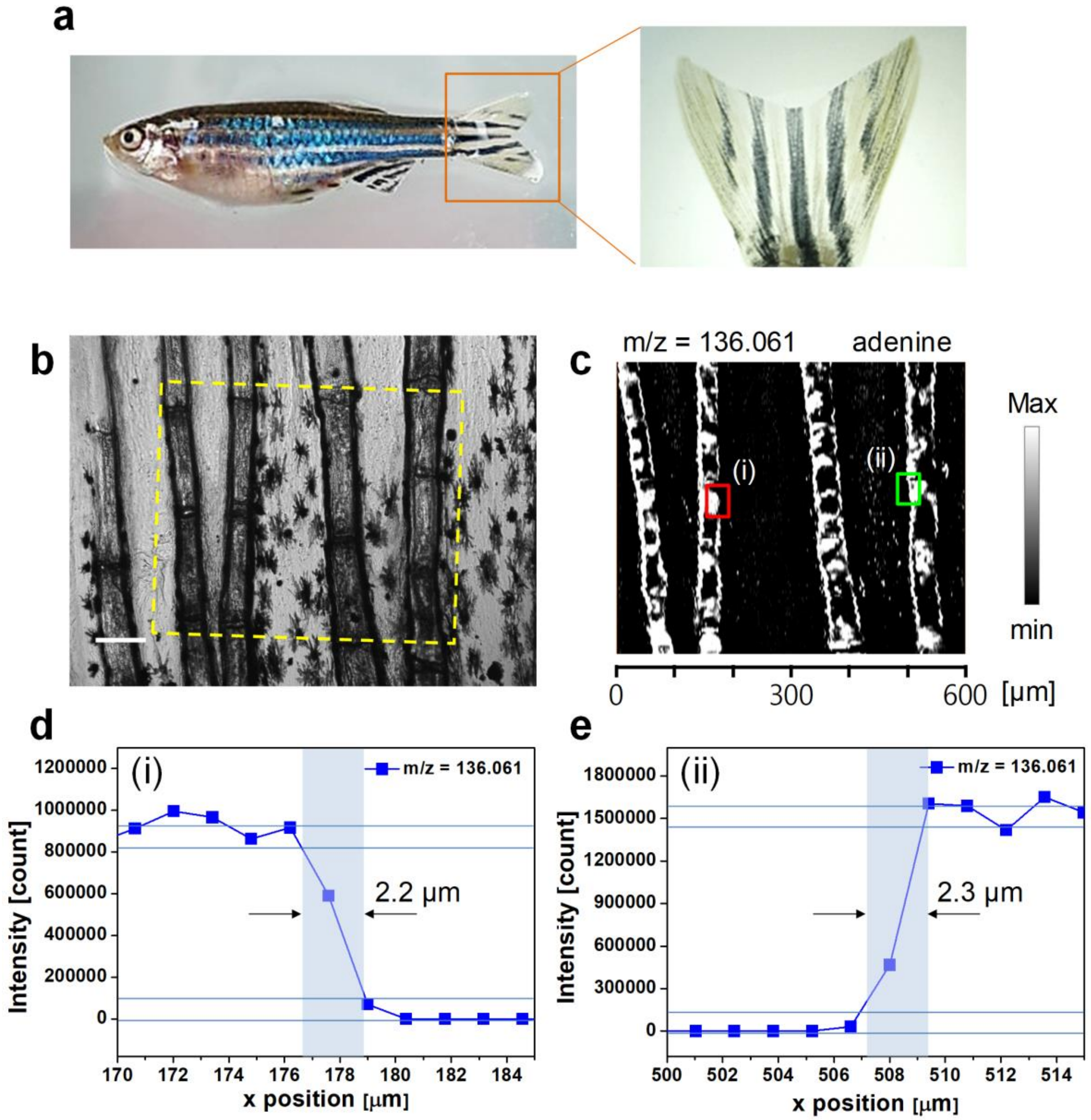

Supplementary Fig. 8 The x-axial lateral resolution test of AP-nanoPALDI imaging in (**a**, **b**) a caudal fin of zebrafish (Danio rerio). Scale bar, 100 μm. (**c**) Ion image for the adenine ion at m/z = 136.061 was taken during the analysis of the caudal fin (the area of 600 μm × 500 μm) when the sample moving velocities were set to 10 μm s^{-1}, respectively. (**d**, **e**) Enlarged graphs of the x-line scan of (i) the falling slope and (ii) the rising slope of the ion image.

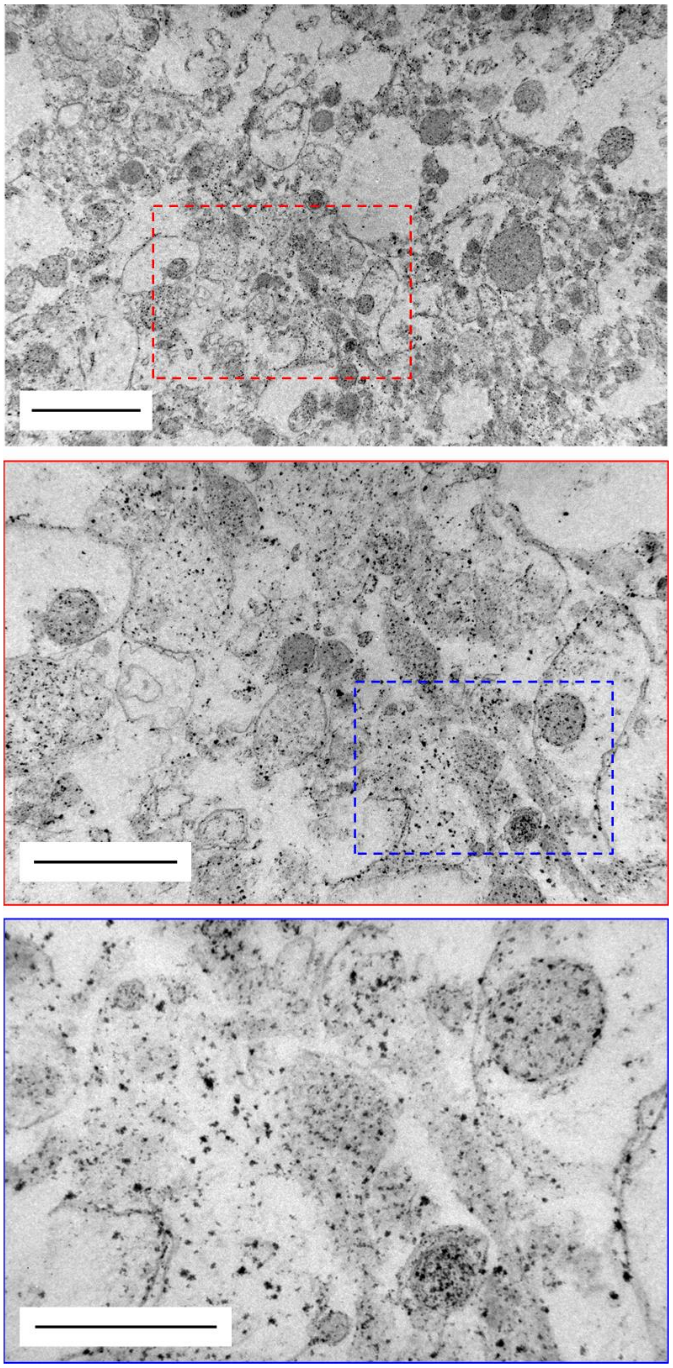

Supplementary Fig. 9 Transmission electron microscopy (TEM) images of the hippocampal tissue slice treated with mPEG-AuNRs for AP-nanoPALDI MS, each using the same tissue slice with different magnifications. TEM images confirmed that mPEG-AuNRs were fairly evenly distributed throughout the tissue. Scale bar, upper: 2 µm, middle: 1 µm, lower: 500 nm.

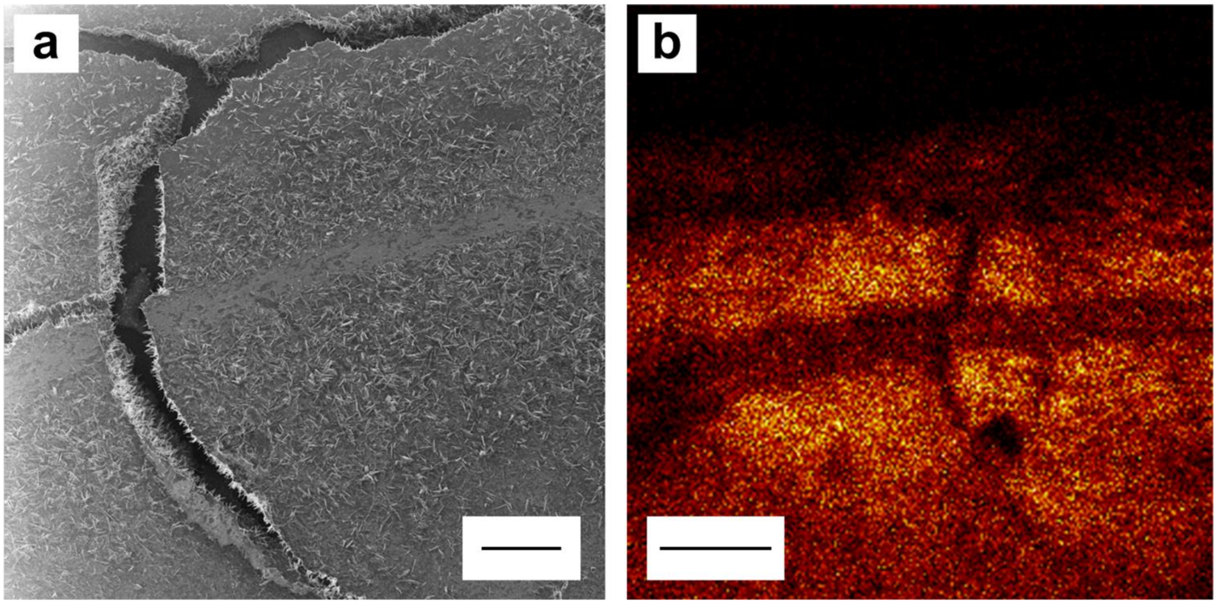

Supplementary Fig. 10 Distortion of original cholesterol distribution measured by vacuum-based secondary ion mass spectrometry (SIMS). (**a**) An HIM image and (**b**) a SIMS image of cholesterol (m/z = 369.3) taken from a hippocampus tissue prepared by slicing and drying procedures. Scale bar, (a) 50 µm and (b) 100 µm. The HIM image shows segregated cholesterol crystals on the hippocampus tissue surface. The HIM image was taken by with an Orion NanoFab Helium Ion Microscope (Carl Zeiss Microscopy, USA) using He^+ ion beam at 35-keV beam energy, with a probe current of ~0.1 pA. TOF-SIMS measurements were carried out with a TOF SIMS V instrument (ION-TOF GmbH, Germany), by using 30-keV Bi_3^+ primary ions. The analysis area of 500 × 500 µm^2 was randomly rastered by the primary ions and was charge-compensated for glass-slide samples by low-energy electron flooding. The primary ion dose density was maintained below 1.0×10^{12} ions cm^{-2} to ensure static SIMS conditions.

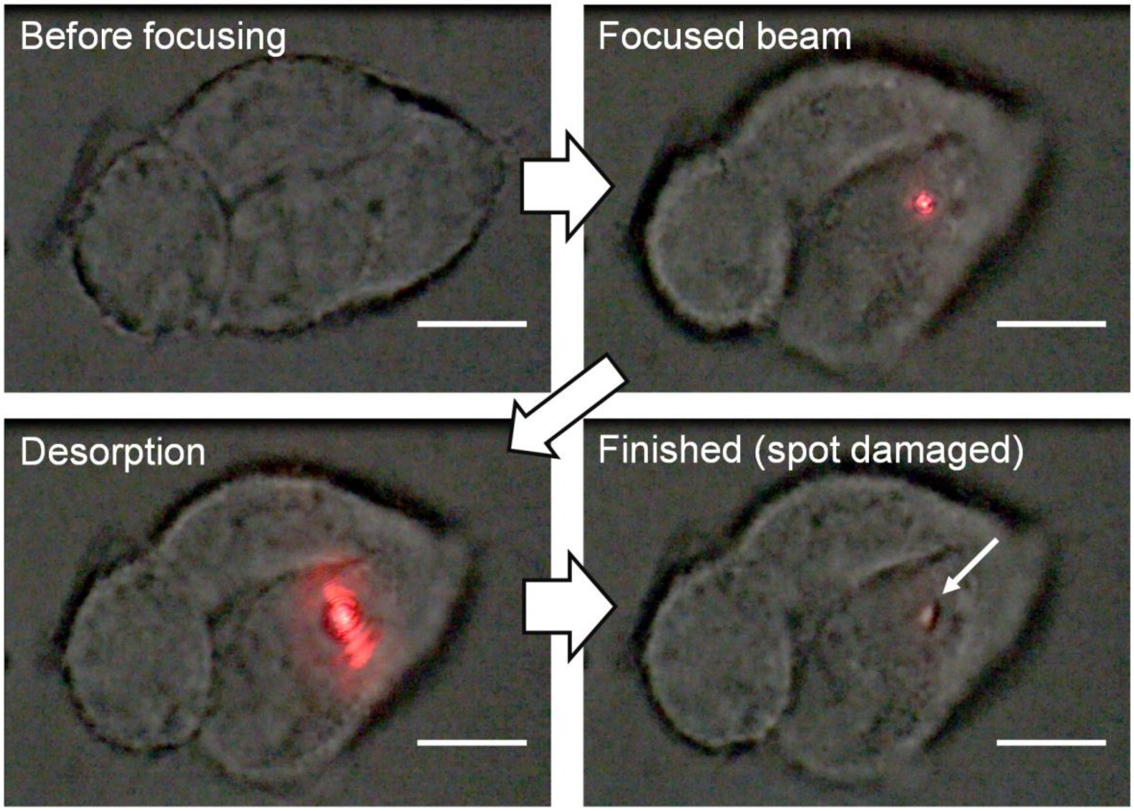

Supplementary Fig. 11 Laser-induced desorption procedure of an HEK cell. When the laser beam focused on one of three HEK cells with objective lens 100×, desorption occurred and spot damage by desorption remained. Scale bar, 5 µm

Supplementary Table

Supplementary Table 1 MS intensities of identified ions with region of interest (ROI)

Molecule<br>m/z		MAG (16:1)<br>311.257		MAG (18:1)<br>339.289		MAG (16:0)<br>313.273		MAG (18:0)<br>341.305		MAG (18:2)<br>337.273	
Region		Apical (a)	Basal (b)	Apical (a)	Basal (b)	Apical (a)	Basal (b)	Apical (a)	Basal (b)	Apical (a)	Basal (b)
Intensity (counts)	ROI 1	98566	69966	610253	414545	1404000	760950	640499	402091	20550	9640
	ROI 2	97467	72980	609936	450191	1426610	852135	638981	433248	22966	10623
	ROI 3	98425	70376	648821	453971	1487400	852381	651979	427409	24795	12528
	Average	98152	71107	623003	439569	1439337	821822	643819	420916	22104	10264
Ratio 1		1	0.72	1	0.71	1	0.57	1	0.65	1	0.48
Ratio 2		1.42	1	1.42	1	1.75	1	1.53	1	2.13	1

Molecule<br>m/z		Cholesterol<br>385.346		Sphingosine<br>282.279		Sphinganine<br>284.295		Ceramide(18:0)<br>548.540	
Region		Apical (a)	Basal (b)	Apical (a)	Basal (b)	Apical (a)	Basal (b)	Apical (a)	Basal (b)
Intensity (counts)	ROI 1	42666	13238	55362	20997	170211	69959	15461	15219
	ROI 2	47378	18185	56556	23775	180696	100567	20353	16841
	ROI 3	55000	18571	70927	25003	200116	123494	19904	19150
	Average	48348	16664	60948	23258	183674	98007	18572	17070
Ratio 1		1	0.35	1	0.38	1	0.53	1	0.92
Ratio 2		2.9	1	2.62	1	1.87	1	1.09	1

Supplementary Note

1. Desorption with small laser beam for high-quality MS imaging

High-quality and high-spatial-resolution MS imaging is closely related to the beam size of lasers for sample desorption. Since a focused laser beam with a higher-magnification objective lens can concentrate its light energy on a smaller area, better desorption behavior can be expected. For instance, when we used a 100× objective lens (UPLSAPO100XO, NA:1.4, Olympus, Japan), diameter of focused laser beam as small as 500 nm could be achieved and laser-induced desorption certainly occurred on a small region, of a diameter less than 1 μm, for a single HEK cell, as shown in **Supplementary Fig. 11**. However, a shallow depth of field caused by high magnification obstructed uniform scanning by laser. Since the live tissue slices sectioned by a tissue chopper and aerated were not ideally flat or uniform in thickness, the laser-induced desorption occurred only in a limited area of the total analysis area, as large as several-hundred to one- or two-thousand square micrometers. Thus, an extremely small-sized laser beam focused by a high magnification lens does not provide a uniform sampling of the whole area, resulting in corrupt MS imaging. In order to secure an appropriate margin of a depth of field for uniform sampling over an entire region of interest (ROI), we "practically" used a 20× objective lens (LUCPLFLN20X, NA:0.45, Olympus, Japan) for focusing, and the resultant diameter of the focused laser beam was 1.2 μm in this study. If the live tissue slices with an identical thickness area can be obtained over the whole sample, a higher-magnification objective lens for focusing the laser beam can be used for submicrometer spatial resolution.

2. Subcellular spatial resolution MS imaging of mouse hippocampal tissue

Since the desorption and ionization were continuously proceeding at atmospheric pressure, the internal analyzing speed (scan speed) of the mass analyzer in the sequence mode strongly influenced how fast and how many mass spectra were acquired, thereby affecting the pixel size of MS imaging. With the optimized parameter settings of the mass analyzer as described in the Methods section, the mass spectra were acquired 433 times per minute (7.2 times per second). In MS imaging achieved by the AP-nanoPALDI method, therefore, the x-axis pixel size (and the corresponding x-axial spatial resolution) was determined by a sample moving speed (*i.e.*, moving speed of the sample stage) with x-axial direction, and the y-axis pixel size and spatial resolution were determined by a distance between two adjacent x-axial laser beam paths. This means that the pixel size of MS imaging could be adjusted according to the size of a region of interest and the total analysis time required. While the sample moving velocity was set to 30 μm s^{-1} to analyze the whole hippocampal region as shown in **Fig. 3**, it was set to μm s^{-1} to analyze the sub-regions of the hippocampal tissue, as shown in **Fig. 4**. Therefore, the corresponding x-axial pixel sizes in MS images were improved from 4.2 μm in the case of **Fig. 3** to 1.4 μm in the case of **Fig. 4**. Besides, as mentioned in the Results section of the main text, the x-axial lateral resolutions of these MS images were 2.9 μm and 5.1 μm when the sample moving velocities were 10 μm s^{-1} and 30 μm s^{-1} (**Fig. 5c,d**), respectively. On the other hand, the spacing between adjacent x-axial lines was fixed to 5 μm in both experiments to keep the similar analysis time, and the y-axial pixel size and spatial resolution was equal to 5 μm in both **Fig. 3** and **Fig. 4**.
